# Invasive ventilation and association with outcomes in heart failure-related cardiogenic shock

**DOI:** 10.1007/s00392-026-02953-z

**Published:** 2026-06-15

**Authors:** Lisa Besch, Caroline Kellner, Jonas Sundermeyer, Benedikt N. Beer, Angela Dettling, Marvin Kriz, Carsten Skurk, Peter Lüdike, Tienush Rassaf, Stefan Kluge, Holger Thiele, Alastair Proudfoot, Tobias Graf, Enzo Lüsebrink, Paulus Kirchhof, Stefan Blankenberg, Benedikt Schrage

**Affiliations:** 1 Department of Cardiology, University Heart and Vascular Center Hamburg, University Medical Center Hamburg-Eppendorf, Martinistr. 52, 20246 Hamburg, Germany; 2 German Centre for Cardiovascular Research (DZHK), Partner Site North, Hamburg, Germany; 3 Institute of Cardiovascular Sciences, University of Birmingham, Birmingham, UK; 4 Department of Cardiology, Angiology and Intensive Care Medicine, Deutsches Herzzentrum der Charité (DHZC), Campus Benjamin Franklin, Berlin, Germany; 5 Franklin/German Centre for Cardiovascular Research (DZHK), Partner Site Berlin/Institute of Health (BIH), Berlin, Germany; 6 Department of Cardiology and Vascular Medicine, West German Heart and Vascular Center, University Hospital Essen, Essen, Germany; 7 Department of Intensive Care Medicine, University Medical Center Hamburg-Eppendorf, Hamburg, Germany; 8 Department of Internal Medicine and Cardiology, Heart Center Leipzig at Leipzig University and Leipzig Heart Science, Leipzig, Germany; 9 Department of Perioperative Medicine, St. Bartholomew’s Hospital, London, UK; 10 University Heart Center Lübeck, University Hospital Schleswig-Holstein, Lübeck, Germany; 11 Department of Clinical Science and Education, Södersjukhuset, Karolinska Institutet, Stockholm, Sweden; 12 Center for Population Health Innovation (POINT), University Heart and Vascular Center Hamburg, University Medical Center Hamburg-Eppendorf, Hamburg, Germany; 13 Medizinische Klinik Und Poliklinik II, University Clinic Bonn, Bonn, Germany; 14 Université Paris Cité and Université Sorbonne Paris Nord, INSERM, INRAE, Centre for Research in Epidemiology and Statistics (CRESS), Paris, France

**Keywords:** Cardiogenic shock, Heart failure, Invasive ventilation, PaO_2_/FiO_2_ quotient

## Abstract

**Background:**

Respiratory failure occurs frequently in cardiogenic shock but with varying severity. It is unknown whether invasive ventilation should be used strictly, reserved for severe respiratory failure, or liberally, reducing stress and increasing compliance with intensive care procedures. Aim of this study was to evaluate the impact of invasive ventilation on outcomes in patients with heart failure-related cardiogenic shock across the severity of respiratory failure.

**Methods:**

Patients with heart failure-related cardiogenic shock treated 2010–2021 in 16 tertiary care centers in five countries were enrolled. Cox regression models were constructed to assess the impact of overall invasive ventilation and invasive ventilation stratified by PaO_2_/FiO_2_ ≤ 100, 101–200, and > 200 on 30-day mortality, adjusted for relevant confounders.

**Results:**

Of *N* = 1010 patients (median age 64 years, 724 (71.7%) male), 659 patients (65.2%) received invasive ventilation. In patients receiving invasive ventilation, 30-day mortality was 1.67 times higher (95% confidence interval (CI) 1.26–2.22, *p* < 0.001) than in patients without invasive ventilation. Although patients with a worse PaO_2_/FiO_2_ of ≤ 100 and 101–200 had the highest mortality risk (hazard ratio (HR) 1.74, 95% CI 1.19–2.53, *p* = 0.004 and HR 1.82, 95% CI 1.29–2.56, *p* < 0.001), the same trend towards increased mortality persisted even in patients with preserved gas exchange and PaO_2_/FiO_2_ > 200 (HR 1.39, 95% CI 0.99–1.97, *p* = 0.06).

**Conclusions:**

In patients with cardiogenic shock, invasive ventilation was associated with higher mortality, with a non-significant trend towards higher mortality even in patients with preserved gas exchange. The results call for further analyses on this topic, specifically to clarify whether dedicated ventilator settings or weaning strategies may improve outcomes for affected patients.

**Graphical abstract:**

**Figure:**
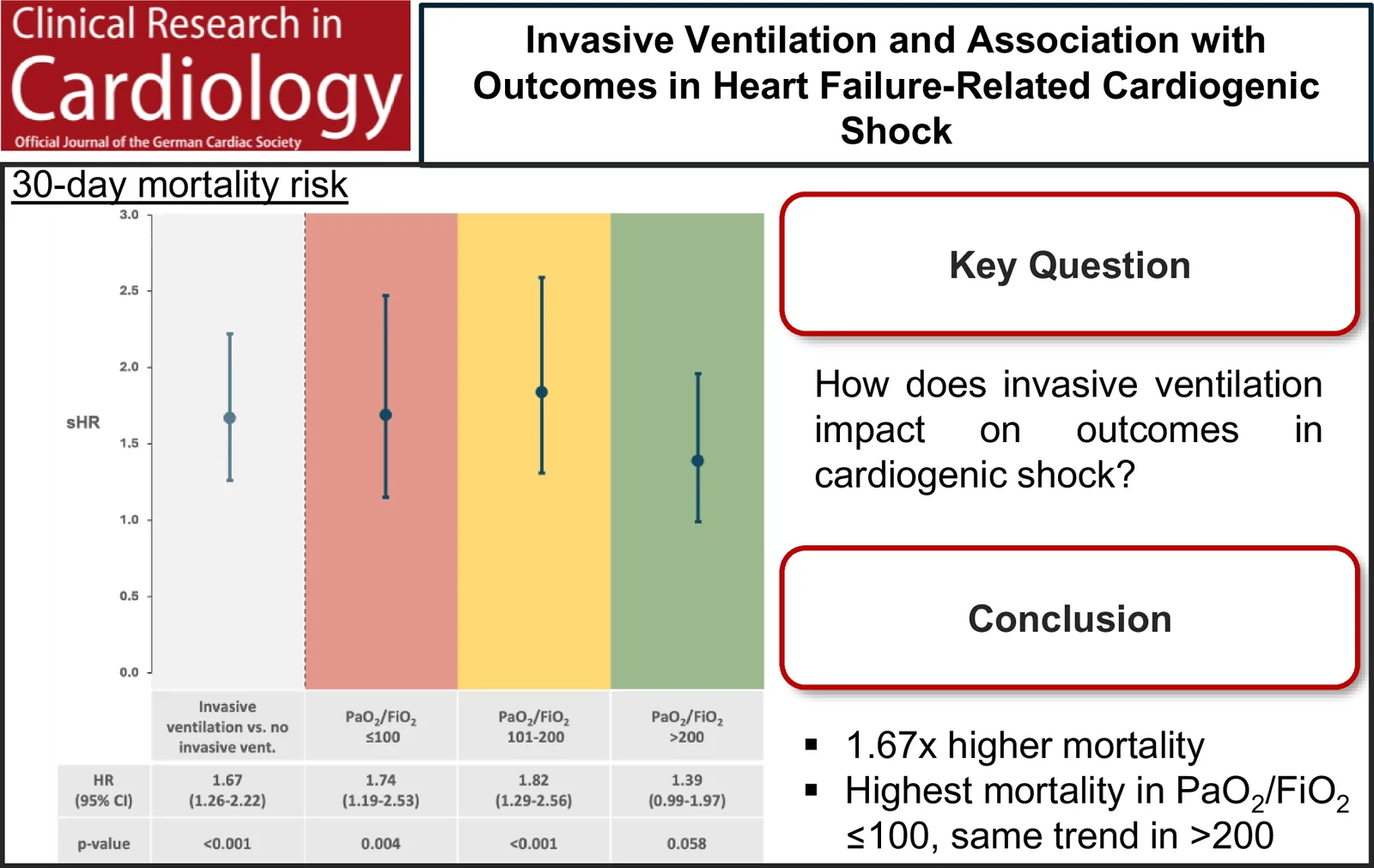

**Supplementary Information:**

The online version contains supplementary material available at https://doi.org/10.1007/s00392-026-02953-z.

## Background

Patients treated for cardiogenic shock often require invasive ventilation [1]. Indications range from protective intubation in case of reduced consciousness (e.g., after cardiac arrest) to electrical storm over to respiratory distress in severe cardiogenic shock [2–4]. Further indications are pulmonary edema/effusion or pneumonia with subsequent respiratory failure [5, 6].

In patients with reduced consciousness due to cerebral hypoperfusion or after cardiac arrest, invasive ventilation may be established for neurologic reasons and not primarily due to impaired gas exchange or lung mechanics. Cardiac arrest with neurological impairment may lead to prolonged invasive ventilation [7].

Cardiogenic shock per se is associated with high mortality and long durations of hospital stay [8, 9]. Moreover, invasive ventilation is also associated with worse outcomes in terms of mortality and length of hospital stays [10, 11]. Invasive ventilation and requisite sedation often entail a reduction in endogenous catecholamine synthesis which may aggravate hemodynamic dysregulation. Further, direct implications of invasive ventilation include barotrauma, muscular weakness, and ventilator-associated pneumonia [12–15]. Respiratory failure and specifically invasive ventilation might therefore not only be a risk mediator but could impact outcomes itself.

The aim of this study was to evaluate the impact of invasive ventilation on outcomes in patients with heart failure-related cardiogenic shock across the severity of respiratory failure.

## Methods

This study was performed in accordance with the Declaration of Helsinki and approved by the local ethics committees (identifier of main committee: PV5607). Due to the retrospective design of the study and analysis of fully anonymized data, the main ethics committee waived the requirement for informed consent.

### Study design

This international observational study enrolled patients with heart failure-related cardiogenic shock between January 2010 and December 2021 in 16 tertiary care centers of five different countries (NCT03313687). The detailed study protocol for this registry has been reported previously [16–18].

Patients included in this analysis suffered from heart failure-related shock resulting from either de novo or acute on chronic heart failure. Patients with acute myocardial infarction or indication for coronary revascularization were excluded. Further exclusion criteria were cardiogenic shock primarily caused by right heart failure (e.g., in acute pulmonary embolism), veno-arterial extracorporeal membrane oxygenation (VA-ECMO)–assisted resuscitation, post-cardiotomy cardiogenic shock, and severe preexisting medical conditions associated with a life expectancy of less than 6 months.

### Study groups

Patients were stratified into the following groups:

1. All patients
2. Patients without invasive ventilation
3. Patients with invasive ventilation

Patients with invasive ventilation were further stratified by the following PaO_2_/FiO_2_ quotient cut-offs at baseline, assessed within 6 h after intubation (worst value within 6 h after intubation):

I. Preserved gas exchange PaO_2_/FiO_2_ > 200
II. Moderate respiratory failure with PaO_2_/FiO_2_ 101–200
III. Severe respiratory failure with PaO_2_/FiO_2_ ≤ 100

### Statistical analysis

For continuous variables, the quartiles are shown as median (1st quartile, 3rd quartile). For binary variables, absolute and relative frequencies are given. Between-group comparisons were done using Kruskal–Wallis test for continuous variables and chi-squared test for binary variables.

Clinical outcomes were duration of ICU stay and 30-day mortality. A multivariable mixed-effects linear regression model with center as random intercept was applied to evaluate the association of invasive ventilation overall as well as invasive ventilation stratified by PaO_2_/FiO_2_ quotient cut-offs with duration of ICU stay. A multivariable center-stratified Cox proportional hazard regression model was applied to evaluate the association of invasive ventilation overall as well as invasive ventilation stratified by PaO_2_/FiO_2_ quotient cut-offs with 30-day mortality.

Additionally, a mixed-effects logistic regression was applied to evaluate the association of invasive ventilation overall and stratified by PaO_2_/FiO_2_ and death during hospital stay.

Models were adjusted for age, sex, body mass index (BMI), prior resuscitation, lowest pH value within 12 h (maximum 6 h before and 6 h after the index event), and highest lactate within 12 h (maximum 6 h before and 6 h after the index event). Reference category was no invasive ventilation, and individuals with missing data for the adjustment variables were excluded from the respective analyses.

The median follow-up time was estimated for mortality by the reverse Kaplan–Meier estimator and mortality rate by Kaplan–Meier method. The results are given as hazard ratios (HR) with their 95% confidence interval (CI).

All computations were performed using R version 4.3.1. Statistical significance is denoted by a two-sided *p*-value < 0.05.

## Results

### Baseline characteristics

Overall, 1010 cardiogenic shock patients were included in the analysis (patient characteristics shown in Table 1). Median age was 64.0 years (interquartile range (IQR) 52.0–75.0), 724 were male (71.7%), 531 patients (52.6%) had an acute on chronic heart failure, and 659 patients (65.2%) were treated with invasive ventilation.

**Table 1:** Baseline characteristics of the cohort

	All patients (*n* = 1010)	No invasive ventilation (*n* = 351)	Invasive ventilation (*n* = 659)	*p*-value
**Demographics**				
Age (years)	64.0 (52.0, 75.0) NAs: 0	66.0 (53.0, 76.0) NAs: 0	64.0 (52.0, 74.0) NAs: 0	0.20
Male no. (%)	724 (71.7) NAs: 0	249 (70.9) NAs: 0	475 (72.1) NAs: 0	0.76
BMI (kg/m^2^)	26.2 (23.3, 30.0) NAs: 39	25.8 (22.2, 29.9) NAs: 12	26.9 (23.9, 30.1) NAs: 27	0.0062
**Comorbidities**				
Arterial hypertension no. (%)	568 (57.4) NAs: 20	198 (56.9) NAs: 3	370 (57.6) NAs: 17	0.88
Diabetes mellitus, any type no. (%)	267 (26.8) NAs: 15	92 (26.5) NAs: 4	175 (27.0) NAs: 11	0.93
Prior coronary revascularization no. (%)	242 (25.1) NAs: 45	86 (25.7) NAs: 16	156 (24.8) NAs: 29	0.82
Known history of heart failure no. (%)	531 (52.6) NAs: 0	215 (61.3) NAs: 0	316 (48.0) NAs: 0	< 0.0001
**Index event**				
Prior resuscitation no. (%)	395 (39.3) NAs: 5	39 (11.2) NAs: 2	356 (54.3) NAs: 3	< 0.0001
PaO_2_/FiO_2_ (worst value within 6 h)	189.0 (102.7, 288.5) NAs: 303	260.0 (143.5, 357.0) NAs: 204	168.7 (100.8, 260.6) NAs: 99	< 0.0001
Creatinine (worst value within 6 h) (mg/dl)	1.7 (1.2, 2.5) NAs: 20	1.7 (1.2, 2.7) NAs: 14	1.6 (1.3, 2.5) NAs: 6	0.92
Systolic blood pressure (worst value within 6 h) (mmHg)	82.0 (70.0, 92.8) NAs: 20	85.0 (75.0, 94.0) NAs: 10	80.0 (70.0, 91.0) NAs: 10	0.00076
Use of vasopressors no. (%)	878 (87.0) NAs: 1	248 (70.7) NAs: 0	630 (95.7) NAs: 1	< 0.0001
Heart rate (worst value within 6 h) (bpm)	96.0 (76.0, 120.0) NAs: 17	100.0 (82.0, 120.0) NAs: 13	93.0 (74.0, 120.0) NAs: 4	0.0016
pH (worst value within 6 h)	7.3 (7.2, 7.4) NAs: 44	7.4 (7.3, 7.4) NAs: 22	7.3 (7.2, 7.4) NAs: 22	< 0.0001
Lactate (worst value within 6 h) (mmol/l)	4.7 (2.5, 8.4) NAs: 46	3.2 (2.2, 6.7) NAs: 23	5.5 (2.8, 9.1) NAs: 23	< 0.0001
Left ventricular ejection fraction (%)	20.0 (15.0, 30.0) NAs: 223	20.0 (15.0, 25.0) NAs: 71	20.0 (15.0, 30.0) NAs: 152	0.20
SCAI: B no. (%)	43 (5.2) NAs: 187	36 (13.4) NAs: 83	7 (1.3) Nas: 104	< 0.0001
SCAI: C no. (%)	316 (38.4) NAs: 187	151 (56.3) NAs: 83	165 (29.7) NAs: 104	< 0.0001
SCAI: D no. (%)	213 (25.9) NAs: 187	42 (15.7) NAs: 83	171 (30.8) NAs: 104	< 0.0001
SCAI: E no. (%)	251 (30.5) NAs: 187	39 (14.6) NAs: 83	212 (38.2) NAs: 104	< 0.0001
**Treatment**				
No mechanical circulatory support no. (%)	607 (60.1) NAs: 0	264 (75.2) NAs: 0	343 (52.0) NAs: 0	< 0.0001

Baseline characteristics of the cohort for the following groups: all patients, patients without invasive ventilation, and patients with invasive ventilation. Missing values are given by NA; furthermore, *p*-values are given

*BMI* body mass index, *SCAI* shock stages defined by the Society for Cardiovascular Angiography & Interventions

Patients treated with invasive ventilation had a higher BMI (26.9 (IQR 23.9–30.1) versus 25.8 kg/m^2^ (IQR 22.2–29.9), *p* = 0.0062), less history of heart failure (48% (*n* = 316) versus 61.3% (*n* = 215), *p* < 0.0001), and were more often resuscitated from cardiac arrest (54.3% (*n* = 356) versus 11.2% (*n* = 39), *p* < 0.0001). In invasively ventilated patients, pH was lower (7.3 (IQR 7.2–7.4) versus 7.4 (IQR 7.3–7.4), *p* < 0.0001) and lactate was higher (5.5 (IQR 2.8–9.1) versus 3.2 (IQR 2.2–6.7), *p* < 0.0001). An additional table on baseline characteristics of the cohort stratified by PaO_2_/FiO_2_ quotient cut-offs is given in the supplement (supplemental Table A).

PaO_2_/FiO_2_ at baseline was 189.0 (IQR 102.7–288.5) in the overall cohort.

### Duration of ICU stay

In patients with invasive ventilation, ICU stay was prolonged by 2.87 days in comparison to patients without invasive ventilation, even after adjusting for relevant confounders (Beta 2.87, 95% CI 0.87–4.88, *p* = 0.005) (Table 2). With regard to PaO_2_/FiO_2_ groups, ICU stay was 3.83 days longer in patients with severe respiratory failure represented by the stratum with the lowest PaO_2_/FiO_2_ quotient (Beta 3.83, 95% CI 0.76–6.9, *p* 0.015) compared to patients without invasive ventilation (Table 2). Notably, invasively ventilated patients with a preserved gas exchange had longer ICU stays in comparison to invasively ventilated patients with moderate respiratory failure (Beta 3.57, 95% CI 1.04–6.1, *p* 0.006 versus Beta 1.96, CI − 0.76 to 4.67, *p* = 0.16). Invasive ventilation was associated with a lower frequency of discharge from ICU (50.8% vs. 70.7%, *p* < 0.0001).

**Table 2:** Association of invasive ventilation with duration of intensive care unit stay in days

	Beta (95% CI)	*p*-value
Invasive ventilation (yes)	2.87 (0.87–4.88)	0.005
Invasive ventilation and PaO_2_/FiO_2_ ≤ 100	3.83 (0.76–6.9)	0.015
Invasive ventilation and PaO_2_/FiO_2_ 101–200	1.96 (− 0.76 to 4.67)	0.16
Invasive ventilation and PaO_2_/FiO_2_ > 200	3.57 (1.04–6.1)	0.006

Duration of intensive care unit stay given in days with the respective confidence intervals, adjusted for age, sex, body mass index, prior resuscitation, pH, and lactate stratified by invasive ventilation and invasive ventilation with the respective PaO_2_/FiO_2_ quotients ≤ 100, 101–200, and > 200

*CI *confidence interval

### Mortality

Overall, 438 patients (overall 30-day mortality rate 51.4%) died. The median follow-up time was 25 days (95% CI 23–27 days) with a 30-day mortality rate of 41.08% in patients without invasive ventilation and 56.48% in patients with invasive ventilation (Table 3).

**Table 3:** Follow-up time and 30-day mortality

	Median follow-up time (95% CI) in days	30-day mortality rate in %	N events 30 days
All patients	25 (23–27)	51.4	438
No invasive ventilation	22 (19.9–25)	41.08	111
Invasive ventilation	27 (24–30)	56.48	327

Follow-up time in days, 30-day mortality rate, and number of events (*N* events) within 30 days are given for all included patients and for patients without and with invasive ventilation

*CI *confidence interval

**Fig. 1:**
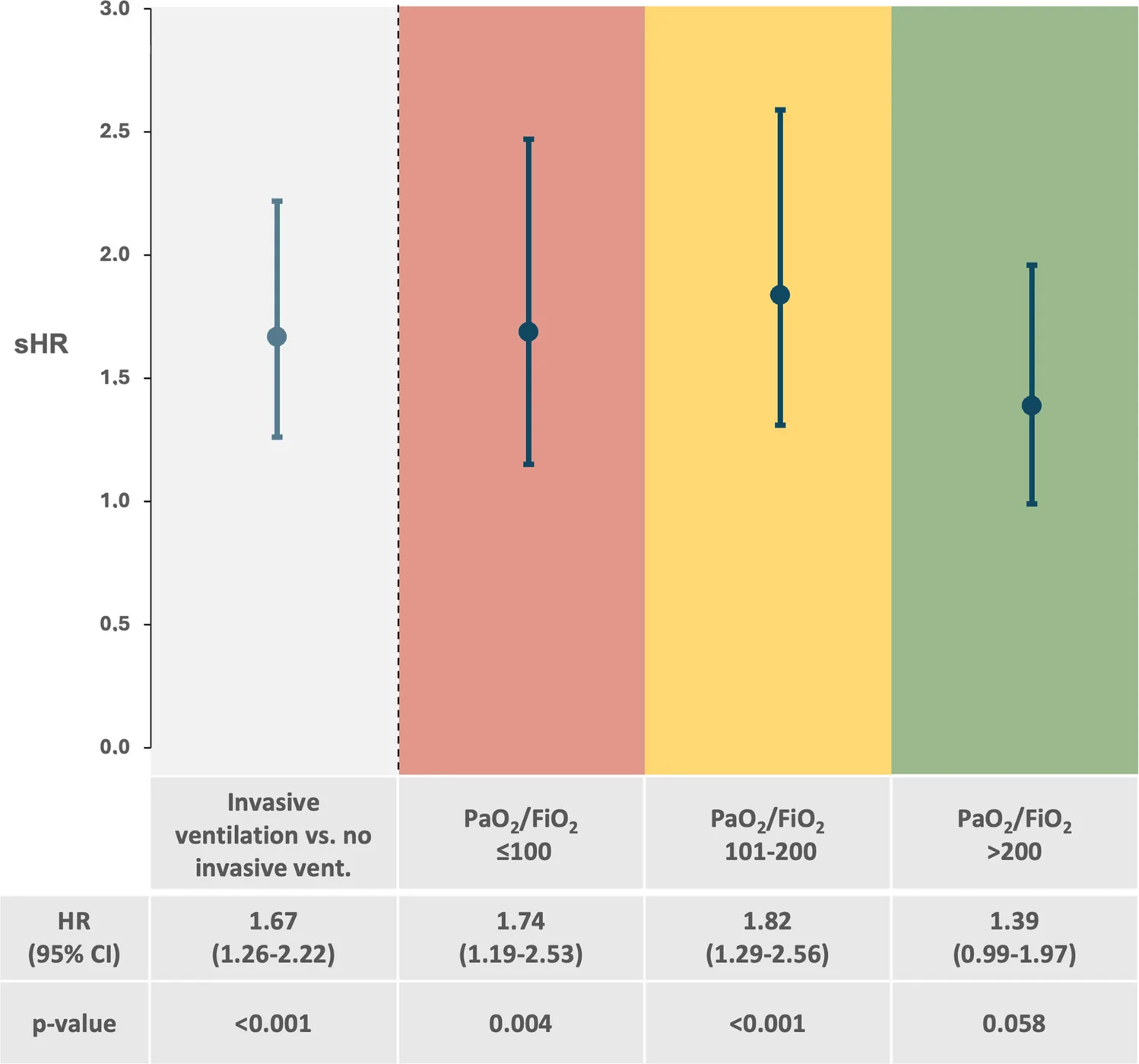
30-day mortality risk in patients with versus without invasive ventilation and stratified by PaO_2_/FiO_2_ quotient. Hazard ratios and 95% confidence intervals for 30-day mortality are given for patients with versus without invasive ventilation (grey) as the reference category and for invasively ventilated patients stratified by PaO_2_/FiO_2_ quotient ≤ 100 (red), 101–200 (yellow), and > 200 (green) versus patients without invasive ventilation. Abbreviations: HR, hazard ratio; CI, confidence interval

In patients with invasive ventilation, likelihood of 30-day mortality was 1.67 times higher (adjusted hazard ratio (aHR) 95% CI 1.26–2.22 *p* < 0.001) than in patients without invasive ventilation after adjusting for relevant confounders (Fig. 1). In addition, odds for death during hospital stay were higher for invasively ventilated patients (adjusted odds ratio 2.56, 95% CI 1.8–3.64, *p* < 0.001).

With regard to 30-day mortality, patients with impaired gas exchange with a PaO_2_/FiO_2_ of ≤ 100 and 101–200 had the highest mortality risk (aHR 1.74, 95% CI 1.19–2.53, *p* = 0.004 and aHR 1.82, 95% CI 1.29–2.56, *p* < 0.001) (Fig. 1). Notably, the trend of a higher mortality persisted even in invasively ventilated patients with a preserved gas exchange and PaO_2_/FiO_2_ > 200 (aHR 1.39, 95% CI 0.99–1.97, *p* 0.06).

## Discussion

In patients with heart failure-related cardiogenic shock, invasive ventilation was associated with longer ICU stay and higher mortality. Importantly, the same trend was observed even in patients with a preserved gas exchange.

These results pose the question if invasive ventilation itself could increase risk of death in patients with cardiogenic shock. Further research into this hypothesis is warranted. Our results support rapid weaning from ventilation in cardiogenic shock.

### Longer ICU stays in patients with invasive ventilation

Invasive ventilation was associated with prolonged ICU stays especially in patients with severe respiratory failure. Interestingly, invasively ventilated patients with a preserved gas exchange had a longer ICU stay compared to patients with moderate respiratory failure, presumably due to more severe cardiogenic shock with generally intact pulmonary function or due to more severe neurologic impairment in patients with cardiac arrest. It can be assumed that the indications for invasive ventilation differed among the three PaO_2_/FiO_2_ groups. Longer ICU stays may thus indicate different complications along the clinical course such as, for example, infection, refractory shock, neurologic impairment, or ongoing respiratory failure requiring prolonged invasive ventilation.

This analysis may be the first larger retrospective analysis on duration of ICU stay in invasively ventilated patients with cardiogenic shock, with and without primarily respiratory failure, stratified by PaO_2_/FiO_2_ quotients. Further studies that also consider the indication for invasive ventilation, neurological status, and both ventilator- and shock-associated complications are warranted.

The weaning approach (direct extubation versus spontaneous breathing trials versus tracheostomy) also influences the duration of ICU stay and furthermore impacts on mortality in critically ill patients treated on intensive care units [19]. Further aspects, such as early wakening, mobilization, and nutrition strategies, may support or hinder weaning processes in invasively ventilated, critically ill patients [20]. Our analysis is the first to imply a similar signal, independent of lung failure, in patients with cardiogenic shock. However, a prospective randomized clinical trial is warranted to compare and assess different ventilation and weaning approaches, sedation and awakening strategies, and forms of nutrition and mobilization in order to derive specific recommendations for this vulnerable population.

### Higher mortality risk in patients with invasive ventilation

Invasive ventilation was associated with higher mortality, with the same trend even in patients with preserved pulmonary function (PaO_2_/FiO_2_ > 200 mmHg). As by gas exchange, the subgroup with a preserved gas exchange would not necessarily require an invasive ventilation strategy; further evaluation of risk factors and management is warranted.

Nonetheless, the results suggest that invasive ventilation could be harmful in patients with cardiogenic shock, either as a risk marker (e.g., indicating other risks leading to the need for invasive ventilation) or a risk factor (e.g., directly contributing to an increased mortality risk). Notably, patients with invasive ventilation despite preserved gas exchange (PaO_2_/FiO_2_ > 200 mmHg) presented with higher lactate levels compared to patients without invasive ventilation (5.2 versus 3.3 mmol/l), indicating higher shock severity in patients requiring invasive ventilation. This may suggest that cardiogenic shock severity represents the primary driver of mortality, while invasive ventilation could act as a contributing factor. One possible explanation is that the alteration in intrathoracic pressure associated with invasive ventilation is affecting hemodynamic parameters [21]. A previous work on invasively ventilated patients demonstrates that higher positive end-expiratory pressure (PEEP) can, for example, lead to a decrease in cardiac index in volume-depleted patients [22]. Currently, studies on hemodynamic assessment in patients with cardiogenic shock with regard to invasive ventilation, however, are limited.

A retrospective analysis of 772 cardiogenic shock patients with assessment of outcomes in non-invasively compared to invasively ventilated patients supports the present results [23]. In that analysis, patients with invasive ventilation had more severe cardiogenic shock, with higher lactate levels and higher mortality at 30 days. Patients with severe respiratory failure (PaO_2_/FiO_2_ < 100 mmHg) had the highest mortality. This finding is consistent with a further study of 1303 patients with moderate to severe acute respiratory distress syndrome (ARDS), in which PaO₂/FiO₂ was assessed at 24 hours. Patients with PaO₂/FiO₂ ≥ 150 mmHg had an ICU mortality of 20%, whereas those with PaO₂/FiO₂ < 150 mmHg had an ICU mortality exceeding 45% [24]. Likewise, in an international, multicenter, prospective cohort study on patients with non-invasive or invasive ventilation due to ARDS, hospital mortality was highest in patients with severe ARDS (46.1% in patients with PaO_2_/FiO_2_ < 100 mmHg versus 40.3% in patients with PaO_2_/FiO_2_ 100 to ≤ 200 and 34.9% in patients with PaO_2_/FiO_2_ 200 to ≤ 300 mmHg) [25].

### Limitations

This hypothesis-generating analysis conducted in a large, international data set of over 1000 patients with cardiogenic shock has several limitations:

The main limitation of this study is the retrospective non-randomized design, so that the results do not allow for conduction of causal relationships between invasive ventilation in cardiogenic shock patients and the respective outcomes, but rather for generation of hypotheses by the associations found. While the analyses were adjusted for known confounders, residual confounding is likely, especially confounding by indication for invasive ventilation, e.g., secondary to brain damage as clinical neurologic assessment scores or, e.g., information on infection or volume status or aspiration were missing in the present data set. Also unmeasured confounders are likely to bias the findings, but we were unable to quantify the strength of unmeasured confounding in the current project. Some variables had missing values, and a differential distribution across patient sub-groups might have introduced a bias and impacted our results. Although invasive ventilation was captured at baseline, which was hospital admission, there is still a chance for immortal times bias, e.g., patients being alive long enough to be intubated, to impact our findings which needs to be considered. Furthermore, there was no information about the timing and duration of invasive ventilation as well as on weaning attempts or strategies, more precisely on potential further confounders such as sedation strategy, early wakening, mobilization, and nutrition, which would have allowed for identification of predictors for impaired outcomes. Our findings regarding length of hospital stay might have been impacted by a competing risk of early death, which needs to be accounted for when interpreting the results. External validation in independent cohorts and eventually randomized controlled trials are warranted. In addition, information on the use of non-invasive ventilation was not recorded, precluding comparisons of ventilation strategies and the subsequent outcomes, especially in patients with preserved gas exchange. Moreover, invasive hemodynamic measurements were not available for analysis. Invasive hemodynamic measurements would have allowed for the evaluation of the impact of invasive ventilation on hemodynamics in cardiogenic shock. Additionally, invasive ventilator settings such as PEEP, driving pressure, or tidal volume were not recorded, and further ventilation-associated information such as the spontaneous breathing fraction, for example, is missing. Thus, the impact of invasive ventilation with regard to ventilator settings on pre- and afterload in cardiogenic shock could not be evaluated. Besides, the right ventricular function was not recorded, which would have enabled a more differentiated analysis of the impact of invasive ventilation on outcomes in patients with left ventricular failure in comparison to right or biventricular failure.

Finally, pulmonary comorbidities were not recorded.

## Conclusions

Invasive ventilation was associated with longer ICU stays and a higher likelihood of death in heart failure-related cardiogenic shock patients. The results suggest that patients receiving invasive ventilation in cardiogenic shock are at higher risk of an impaired prognosis, potentially even patients with invasive ventilation despite a preserved pulmonary function, in whom we observed a non-significant trend towards a higher mortality risk. Invasive ventilation indicated higher shock severity and needs to be further evaluated as a risk marker or a disease modifier. These results call for further research on different ventilation, weaning, and sedation strategies in this vulnerable population.

## Supplementary Information

Below is the link to the electronic supplementary material.

Supplementary file1 (DOCX 27 KB)

## Abbreviations

ARDS: Acute respiratory distress syndrome
RV: Right ventricle
LV: Left ventricle
SCAI stage: Shock stages defined by the Society for Cardiovascular Angiography & Interventions
MCS: Mechanical assist devices
ICU: Intensive care unit
VA-ECMO: Veno-arterial extracorporeal membrane oxygenation
BMI: Body mass index
IQR: Interquartile range
CI: Confidence interval
HR: Hazard ratio
OR: Odds ratio
