# Supplementary material for: Invasive ventilation and association with outcomes in heart failure-related cardiogenic shock
Source: Clin Res Cardiol. 2026 Jun 15;115(11):2015–23. doi: 10.1007/s00392-026-02953-z (PMC13658319; doi:10.1007/s00392-026-02953-z)
Supplement: Supplementary file 1 [file 392_2026_2953_MOESM1_ESM.docx]

# **Supplementary material**

**Tables**

**Table A: Baseline characteristics** **of the cohort**

|  | **All patients (n=911)** | **No invasive ventilation (n=351)** | **Invasive ventilation and PaO2/FiO2 >100-200 (n=186)** | **Invasive ventilation and PaO2/FiO2<=100 (n=140)** | **Invasive ventilation and PaO2/FiO2>200 (n=234)** | **p-value** |
| --- | --- | --- | --- | --- | --- | --- |
| **Demographics** |  |  |  |  |  |  |
| **Age (Years)** | 64.0 (52.0, 75.0) NAs:0 | 66.0 (53.0, 76.0) NAs:0 | 63.0 (51.2, 73.0) NAs:0 | 68.0 (55.8, 76.0) NAs:0 | 61.0 (52.0, 74.0) NAs:0 | 0.060 |
| **Male No. (%)** | 640 (70.3) NAs:0 | 249 (70.9) NAs:0 | 129 (69.4) NAs:0 | 109 (77.9) NAs:0 | 153 (65.4) NAs:0 | 0.083 |
| **BMI (kg/m^2^)** | 26.3 (23.3, 30.4) NAs:33 | 25.8 (22.2, 29.9) NAs:12 | 26.9 (24.0, 31.0) NAs:8 | 27.8 (24.2, 31.2) NAs:5 | 26.2 (23.9, 30.0) NAs:8 | 0.0042 |
| **Comorbidities** |  |  |  |  |  |  |
| **Arterial hypertension No. (%)** | 513 (57.5) NAs:19 | 198 (56.9) NAs:3 | 106 (59.6) NAs:8 | 82 (59.9) NAs:3 | 127 (55.5) NAs:5 | 0.79 |
| **Diabetes mellitus, any type No. (%)** | 241 (26.9) NAs:14 | 92 (26.5) NAs:4 | 48 (26.5) NAs:5 | 46 (32.9) NAs:0 | 55 (24.0) NAs:5 | 0.32 |
| **Atrial fibrillation, any type No. (%)** | 391 (44.0) NAs:23 | 162 (47.2) NAs:8 | 79 (43.9) NAs:6 | 60 (44.4) NAs:5 | 90 (39.1) NAs:4 | 0.30 |
| **Prior coronary revascularization No. (%)** | 216 (24.9) NAs:42 | 86 (25.7) NAs:16 | 39 (21.9) NAs:8 | 46 (34.6) NAs:7 | 45 (20.2) NAs:11 | 0.016 |
| **Known history of heart failure No. (%)** | 476 (52.3) NAs:0 | 215 (61.3) NAs:0 | 76 (40.9) NAs:0 | 71 (50.7) NAs:0 | 114 (48.7) NAs:0 | <0.0001 |
| **Type of heart failure: HFmrEF No. (%)** | 55 (10.9) NAs:405 | 18 (8.2) NAs:131 | 12 (15.0) NAs:106 | 11 (13.8) NAs:60 | 14 (11.1) NAs:108 | 0.29 |
| **Type of heart failure: HFpEF No. (%)** | 29 (5.7) NAs:405 | 17 (7.7) NAs:131 | 2 (2.5) NAs:106 | 2 (2.5) NAs:60 | 8 (6.3) NAs:108 | 0.19 |
| **Type of heart failure: HFrEF No. (%)** | 422 (83.4) NAs:405 | 185 (84.1) NAs:131 | 66 (82.5) NAs:106 | 67 (83.8) NAs:60 | 104 (82.5) NAs:108 | 0.98 |
| **Most likely cause of heart failure: DCM No. (%)** | 166 (40.4) NAs:500 | 79 (47.6) NAs:185 | 27 (31.0) NAs:99 | 19 (40.4) NAs:93 | 41 (36.9) NAs:123 | 0.063 |
| **Most likely cause of heart failure: ICM No. (%)** | 92 (22.4) NAs:500 | 32 (19.3) NAs:185 | 25 (28.7) NAs:99 | 14 (29.8) NAs:93 | 21 (18.9) NAs:123 | 0.16 |
| **Most likely cause of heart failure: Myocarditis No. (%)** | 45 (10.9) NAs:500 | 14 (8.4) NAs:185 | 9 (10.3) NAs:99 | 4 (8.5) NAs:93 | 18 (16.2) NAs:123 | 0.21 |
| **Most likely cause of heart failure: Other CM No. (%)** | 108 (26.3) NAs:500 | 41 (24.7) NAs:185 | 26 (29.9) NAs:99 | 10 (21.3) NAs:93 | 31 (27.9) NAs:123 | 0.67 |
| **Index Event** |  |  |  |  |  |  |
| **Prior resuscitation No. (%)** | 367 (40.5) NAs:5 | 39 (11.2) NAs:2 | 104 (56.2) NAs:1 | 91 (65.5) NAs:1 | 133 (57.1) NAs:1 | <0.0001 |
| **Duration of resuscitation: <10 min No. (%)** | 196 (45.6) NAs:481 | 65 (85.5) NAs:275 | 41 (37.3) NAs:76 | 26 (29.2) NAs:51 | 64 (41.3) NAs:79 | <0.0001 |
| **Duration of resuscitation: 10-30 min No. (%)** | 154 (35.8) NAs:481 | 4 (5.3) NAs:275 | 43 (39.1) NAs:76 | 40 (44.9) NAs:51 | 67 (43.2) NAs:79 | <0.0001 |
| **Duration of resuscitation: 31-45 min No. (%)** | 39 (9.1) NAs:481 | 6 (7.9) NAs:275 | 12 (10.9) NAs:76 | 10 (11.2) NAs:51 | 11 (7.1) NAs:79 | 0.61 |
| **Duration of resuscitation: >45 min No. (%)** | 41 (9.5) NAs:481 | 1 (1.3) NAs:275 | 14 (12.7) NAs:76 | 13 (14.6) NAs:51 | 13 (8.4) NAs:79 | 0.017 |
| **PaO2/FiO2 (worst value within 6 hours)** | 189.0 (102.7, 288.5) NAs:204 | 260.0 (143.5, 357.0) NAs:204 | 139.8 (121.0, 168.2) NAs:0 | 75.0 (61.7, 88.0) NAs:0 | 286.8 (232.8, 355.8) NAs:0 | <0.0001 |
| **Creatinine (worst value within 6 hours) (mg/dl)** | 1.6 (1.2, 2.5) NAs:15 | 1.7 (1.2, 2.7) NAs:14 | 1.5 (1.2, 2.4) NAs:0 | 1.8 (1.3, 2.6) NAs:1 | 1.6 (1.2, 2.3) NAs:0 | 0.10 |
| **Systolic blood pressure (worst value within 6 hours) (mmHg)** | 81.5 (70.0, 92.0) NAs:17 | 85.0 (75.0, 94.0) NAs:10 | 82.5 (70.0, 95.0) NAs:2 | 80.0 (66.0, 90.0) NAs:2 | 82.0 (70.0, 90.0) NAs:3 | 0.00013 |
| **Diastolic blood pressure (worst value within 6 hours) (mmHg)** | 50.0 (40.0, 57.0) NAs:22 | 50.0 (41.5, 60.0) NAs:12 | 50.0 (41.0, 55.0) NAs:3 | 45.0 (37.5, 52.0) NAs:2 | 50.0 (40.0, 58.0) NAs:5 | 0.00024 |
| **Mean arterial pressure (worst value within 6 hours) (mmHg)** | 61.0 (53.0, 70.0) NAs:321 | 63.5 (55.0, 72.0) NAs:125 | 59.0 (52.0, 67.0) NAs:73 | 59.0 (50.0, 67.2) NAs:57 | 60.0 (50.8, 68.0) NAs:66 | 0.00041 |
| **Use of vasopressors No. (%)** | 782 (85.8) NAs:0 | 248 (70.7) NAs:0 | 180 (96.8) NAs:0 | 132 (94.3) NAs:0 | 222 (94.9) NAs:0 | <0.0001 |
| **Heart rate (worst value within 6 hours) (bpm)** | 96.0 (77.0, 120.0) NAs:16 | 100.0 (82.0, 120.0) NAs:13 | 90.0 (71.0, 120.0) NAs:1 | 96.0 (76.0, 128.0) NAs:1 | 95.0 (75.0, 119.0) NAs:1 | 0.014 |
| **pH (worst value within 6 hours)** | 7.3 (7.2, 7.4) NAs:33 | 7.4 (7.3, 7.4) NAs:22 | 7.3 (7.2, 7.4) NAs:4 | 7.2 (7.1, 7.3) NAs:5 | 7.3 (7.2, 7.4) NAs:2 | <0.0001 |
| **Lactate (worst value within 6 hours) (mmol/l)** | 4.9 (2.5, 8.5) NAs:36 | 3.2 (2.2, 6.7) NAs:23 | 5.8 (2.8, 9.5) NAs:4 | 7.2 (4.3, 10.0) NAs:6 | 5.2 (2.7, 8.5) NAs:3 | <0.0001 |
| **Arterial pH (worst value within 6 hours)** | 7.3 (7.2, 7.4) NAs:51 | 7.4 (7.3, 7.4) NAs:40 | 7.3 (7.2, 7.4) NAs:4 | 7.2 (7.1, 7.3) NAs:5 | 7.3 (7.2, 7.4) NAs:2 | <0.0001 |
| **Arterial lactate (worst value within 6 hours) (mmol/l)** | 5.0 (2.5, 8.6) NAs:57 | 3.3 (2.2, 6.9) NAs:42 | 5.8 (2.8, 9.5) NAs:5 | 7.2 (4.3, 10.0) NAs:6 | 5.2 (2.7, 8.5) NAs:4 | <0.0001 |
| **Left ventricular ejection fraction (%)** | 20.0 (15.0, 30.0) NAs:194 | 20.0 (15.0, 25.0) NAs:71 | 20.0 (15.0, 33.0) NAs:45 | 25.0 (15.0, 32.0) NAs:39 | 20.0 (15.0, 30.0) NAs:39 | 0.095 |
| **SCAI: B No. (%)** | 39 (5.2) NAs:164 | 36 (13.4) NAs:83 | 2 (1.2) NAs:22 | 0 (0) NAs:20 | 1 (0.5) NAs:39 | <0.0001 |
| **SCAI: C No. (%)** | 304 (40.7) NAs:164 | 151 (56.3) NAs:83 | 60 (36.6) NAs:22 | 20 (16.7) NAs:20 | 73 (37.4) NAs:39 | <0.0001 |
| **SCAI: D No. (%)** | 194 (26.0) NAs:164 | 42 (15.7) NAs:83 | 44 (26.8) NAs:22 | 39 (32.5) NAs:20 | 69 (35.4) NAs:39 | <0.0001 |
| **SCAI: E No. (%)** | 210 (28.1) NAs:164 | 39 (14.6) NAs:83 | 58 (35.4) NAs:22 | 61 (50.8) NAs:20 | 52 (26.7) NAs:39 | <0.0001 |
| **Treatment** |  |  |  |  |  |  |
| **No mechanical circulatory support No. (%)** | 555 (60.9) NAs:0 | 264 (75.2) NAs:0 | 101 (54.3) NAs:0 | 77 (55.0) NAs:0 | 113 (48.3) NAs:0 | <0.0001 |
| **Only Impella No. (%)** | 140 (15.4) NAs:0 | 47 (13.4) NAs:0 | 27 (14.5) NAs:0 | 24 (17.1) NAs:0 | 42 (17.9) NAs:0 | 0.44 |
| **Only VA-ECMO No. (%)** | 143 (15.7) NAs:0 | 23 (6.6) NAs:0 | 42 (22.6) NAs:0 | 25 (17.9) NAs:0 | 53 (22.6) NAs:0 | <0.0001 |
| **Impella + VA-ECMO No. (%)** | 73 (8.0) NAs:0 | 17 (4.8) NAs:0 | 16 (8.6) NAs:0 | 14 (10.0) NAs:0 | 26 (11.1) NAs:0 | 0.034 |
| **Complications** |  |  |  |  |  |  |
| **Renal replacement therapy No. (%)** | 283 (31.1) NAs:2 | 95 (27.1) NAs:1 | 67 (36.0) NAs:0 | 51 (36.7) NAs:1 | 70 (29.9) NAs:0 | 0.077 |
| **Sepsis (SIRS criteria and >=2 positive blood cultures) No. (%)** | 156 (17.2) NAs:3 | 42 (12.0) NAs:0 | 39 (21.0) NAs:0 | 39 (28.3) NAs:2 | 36 (15.5) NAs:1 | 0.00011 |
| **Pulmonary oedema No. (%)** | 376 (42.4) NAs:25 | 106 (31.7) NAs:17 | 101 (54.6) NAs:1 | 78 (57.4) NAs:4 | 91 (39.4) NAs:3 | <0.0001 |
| **Hypoxic brain damage on CT No. (%)** | 68 (7.8) NAs:35 | 1 (0.3) NAs:15 | 27 (15.1) NAs:7 | 13 (9.6) NAs:4 | 27 (12.0) NAs:9 | <0.0001 |
| **Outcomes** |  |  |  |  |  |  |
| **Discharge from ICU No. (%)** | 522 (57.3) NAs:0 | 248 (70.7) NAs:0 | 88 (47.3) NAs:0 | 53 (37.9) NAs:0 | 133 (56.8) NAs:0 | <0.0001 |
| **Duration of ICU stay (days)** | 7.8 (3.0, 15.0) NAs:3 | 6.0 (2.9, 13.0) NAs:0 | 7.4 (3.0, 14.9) NAs:1 | 8.1 (1.7, 16.0) NAs:0 | 10.0 (4.8, 16.7) NAs:2 | 0.00046 |
| **Discharge from hospital No. (%)** | 491 (53.9) NAs:0 | 230 (65.5) NAs:0 | 84 (45.2) NAs:0 | 50 (35.7) NAs:0 | 127 (54.3) NAs:0 | <0.0001 |
| **Duration of hospital stay (days)** | 14.0 (7.0, 27.0) NAs:4 | 14.4 (7.7, 27.0) NAs:1 | 12.2 (6.0, 26.0) NAs:2 | 10.9 (3.3, 24.0) NAs:0 | 15.5 (8.0, 29.9) NAs:1 | 0.0064 |
| **Left ventricular ejection fraction at discharge (%)** | 30.0 (20.0, 45.0) NAs:483 | 25.0 (20.0, 40.0) NAs:150 | 35.0 (22.5, 48.0) NAs:123 | 30.0 (24.0, 43.5) NAs:97 | 30.0 (20.0, 45.0) NAs:113 | 0.062 |

Baseline characteristics of the cohort are given for the following groups: All patients, patients without invasive ventilation and patients with invasive ventilation stratified by PaO_2_/FiO_2_ quotient: Preserved gas exchange with PaO_2_/FiO_2_ >200, moderate respiratory failure PaO_2_/FiO_2_ 101-200 and severe respiratory failure PaO_2_/FiO_2_ ≤ 100. Missing values are given by NA, furthermore p-values are given. Abbreviations: Body mass index (BMI), heart failure with mildly reduced ejection fraction (HFmrEF), heart failure with preserved ejection fraction (HFpEF), heart failure with reduced ejection fraction (HFrEF), dilated cardiomyopathy (DCM), ischemic cardiomyopathy (ICM), cardiomyopathy (CM), shock stage according to classification by the Society for Cardiovascular Angiography and Interventions (SCAI B-E), veno-arterial extracorporeal membrane oxygenation (VA-ECMO).

**Table B: Association of invasive ventilation with duration of intensive care unit stay in days, unadjusted.**

|  | **Beta (95% CI)** | **p-value** |
| --- | --- | --- |
| **Invasive ventilation (yes)** | 2.46 (0.76-4.16) | 0.0046 |
| **Invasive ventilation and PaO_2_/FiO_2_** ≤**100** | 2.71 (0.16-5.25) | 0.038 |
| **Invasive ventilation and PaO_2_/FiO_2_ 101-200** | 1.3 (-1.04-3.65) | 0.28 |
| **Invasive ventilation and PaO_2_/FiO_2_ >200** | 3.2 (1.0-5.4) | 0.0045 |

Outcome duration of intensive care unit stay in days given for patients with invasive ventilation and stratified by PaO_2_/FiO_2_ groups, unadjusted. Abbreviations: Confidence interval (CI).

**Table C: 30-day mortality risk in patients with invasive ventilation and with invasive ventilation stratified by PaO_2_/FiO_2_ groups, unadjusted.**

|  | **HR (95% CI)** | **p-value** |
| --- | --- | --- |
| **Invasive ventilation (yes)** | 1.70 (1.35-2.16) | <0.001 |
| **Invasive ventilation and PaO_2_/FiO_2_** ≤**100** | 2.15 (1.59-2.92) | <0.001 |
| **Invasive ventilation and PaO_2_/FiO_2_ 101-200** | 1.96 (1.46-2.92) | <0.001 |
| **Invasive ventilation and PaO_2_/FiO_2_ >200** | 1.34 (1.00-1.80) | 0.047 |

30-day mortality risk is given by the respective hazard ratios in patients with invasive ventilation and invasive ventilation stratified by PaO_2_/FiO_2_ groups, unadjusted. Abbreviations: Hazard ratio (HR), Confidence interval (CI).
